# Supplementary material for: N6-methyladenosine regulated FGFR4 attenuates ferroptotic cell death in recalcitrant HER2-positive breast cancer
Source: Nat Commun. 2022 May 13;13:2672. doi: 10.1038/s41467-022-30217-7 (PMC9106694; doi:10.1038/s41467-022-30217-7)
Supplement: Supplementary file 1 — Supplementary Information [file 41467_2022_30217_MOESM1_ESM.pdf]

Supplementary materials

N6-methyladenosine regulated FGFR4 attenuates ferroptotic cell death in recalcitrant HER2-positive breast cancer

Supplementary Figures

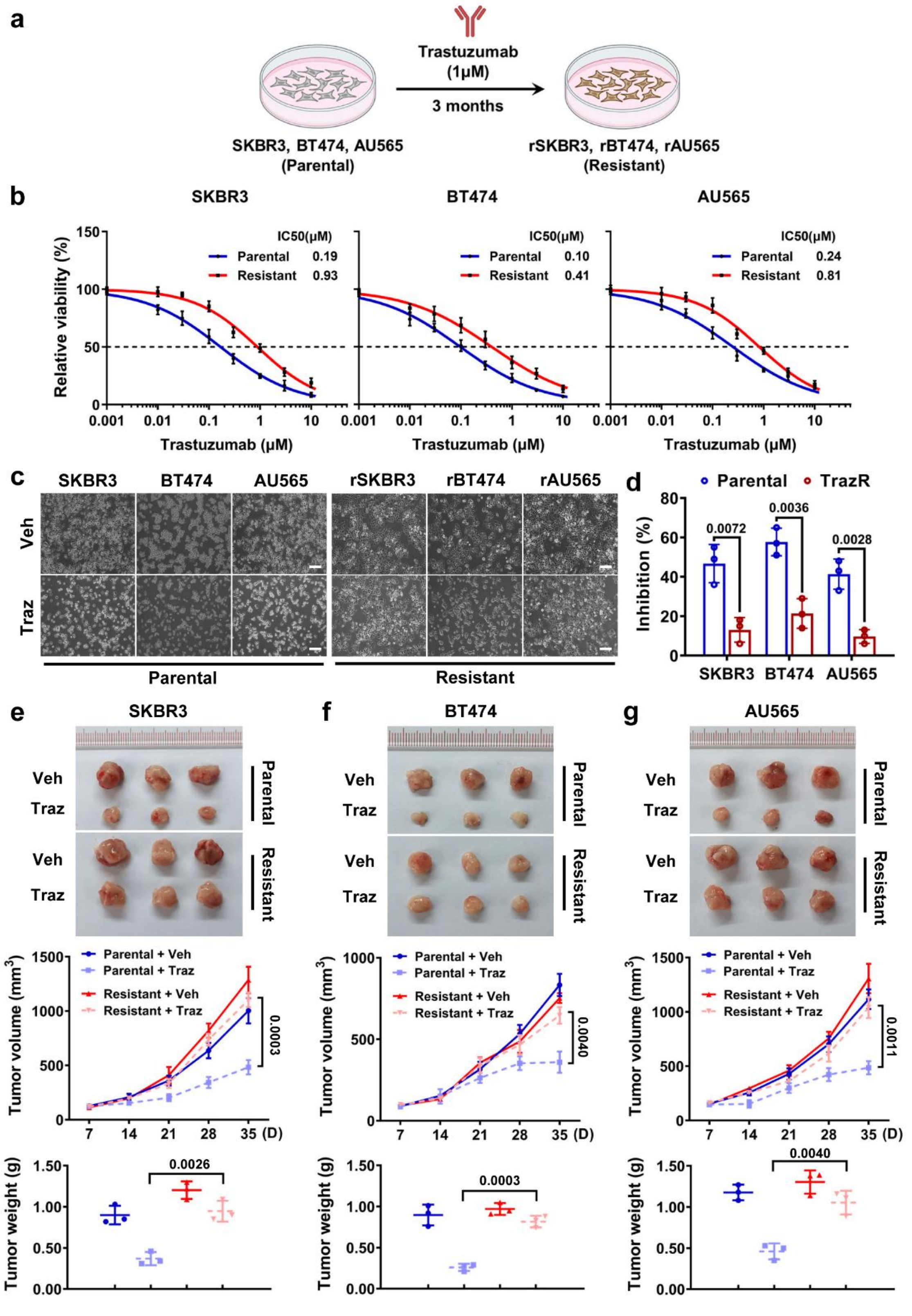

Supplementary Figure 1. Establish and validation of anti-HER2 resistant breast cancer cell lines. **(a)** Parental anti-HER2 sensitive HER2-positive breast cancer cell lines were continuously exposed to trastuzumab for three months to establish anti-HER2 resistant cells. **(b)** Dose-response curves of parental (SKBR3, BT474, and AU565) and resistant cells (rSKBR3, rBT474, and rAU565) after treated with

trastuzumab. Cell viability was measured using absorbance value at 450 nm, determined by CCK-8 assays. Group treated with vehicle (control) was defined as 100% relative viability. **(c)** Bright-field micrographs of cultured parental cells (SKBR3, BT474, and AU565) and cultured resistant cells (rSKBR3, rBT474, and rAU565) treated with vehicle or trastuzumab (0.1 μM) for 24 hours. The resistant cells were withdrawn from trastuzumab for 4 weeks before the experiments. Scale bar=200 μm. **(d)** Inhibitory effect of trastuzumab on parental cells (SKBR3, BT474, and AU565) and resistant cells (rSKBR3, rBT474, and rAU565) was evaluated after treated with trastuzumab (0.1 μM) for 72 hours. The resistant cells were withdrawn from trastuzumab for 4 weeks before the experiments. CCK-8 assay was conducted to evaluate the cell viability. **(e-g)** Tumor-bearing mice established by parental (SKBR3, BT474, and AU565) and resistant cells (rSKBR3, rBT474, and rAU565) were treated with vehicle or trastuzumab (20 mg/kg, intraperitoneal administration). The volume of the tumor was recorded every 7 days, and the tumor growth curves were plotted. Tumors were resected and weighed at the end of the experiment. Data in b, d, e, f, and g were presented as mean ± S.D., n = 3 biologically independent samples. Data were analyzed by two-sided Student's t-test in d-g. Source data are provided as a Source Data file.

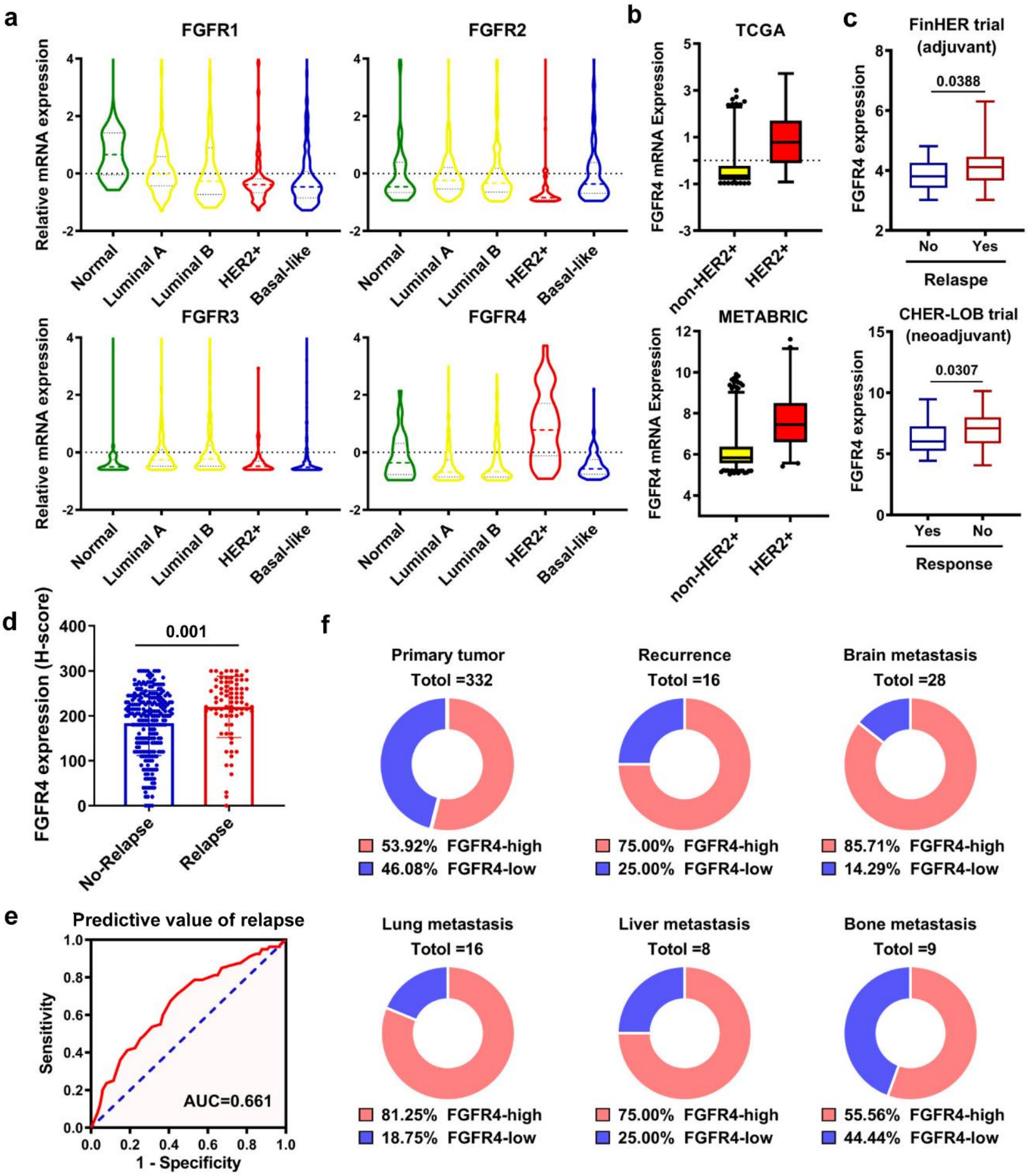

Supplementary Figure 2. FGFR4 was highly expressed in HER2-positive breast cancer and associated with worse survival outcomes.

(a) Expression level of FGFR family members in different molecular subtypes of breast cancer in TCGA cohort. Except of the high expression of FGFR4 in HER2-positive breast cancer, FGFR family members have very low expression levels in breast cancer. **(b)** *FGFR4* mRNA expression in HER2-positive (TCGA n=78, METABRIC n=220) and non-HER2-positive (TCGA n=867, METABRIC n=1538) breast cancer. All boxplots indicate median (center), 25th and 75th percentiles (bounds of box), and minimum and maximum (whiskers). **(c)** *FGFR4* expression in HER2-positive breast cancer patients recruited in FinHER trial (group n=102) and CHER-LOB trial (group n=88). The expression of *FGFR4* was analyzed from

tumors prior to treatment. All boxplots indicate median (center), 25th and 75th percentiles (bounds of box), and minimum and maximum (whiskers). Data were analyzed by two-sided Student's t-test. **(d)** The expression of FGFR4 in specimens of HER2-positive breast cancer with (n=80) or without (n=252) relapse in SYSUCC cohort. Data were analyzed by log-rank test. **(e)** Receiver operating characteristic curve depicts the accuracy of FGFR4 expression in predicting relapse after trastuzumab-based adjuvant therapy. **(f)** Proportion of FGFR4 high expression in various types of samples in breast cancer. Source data are provided as a Source Data file.

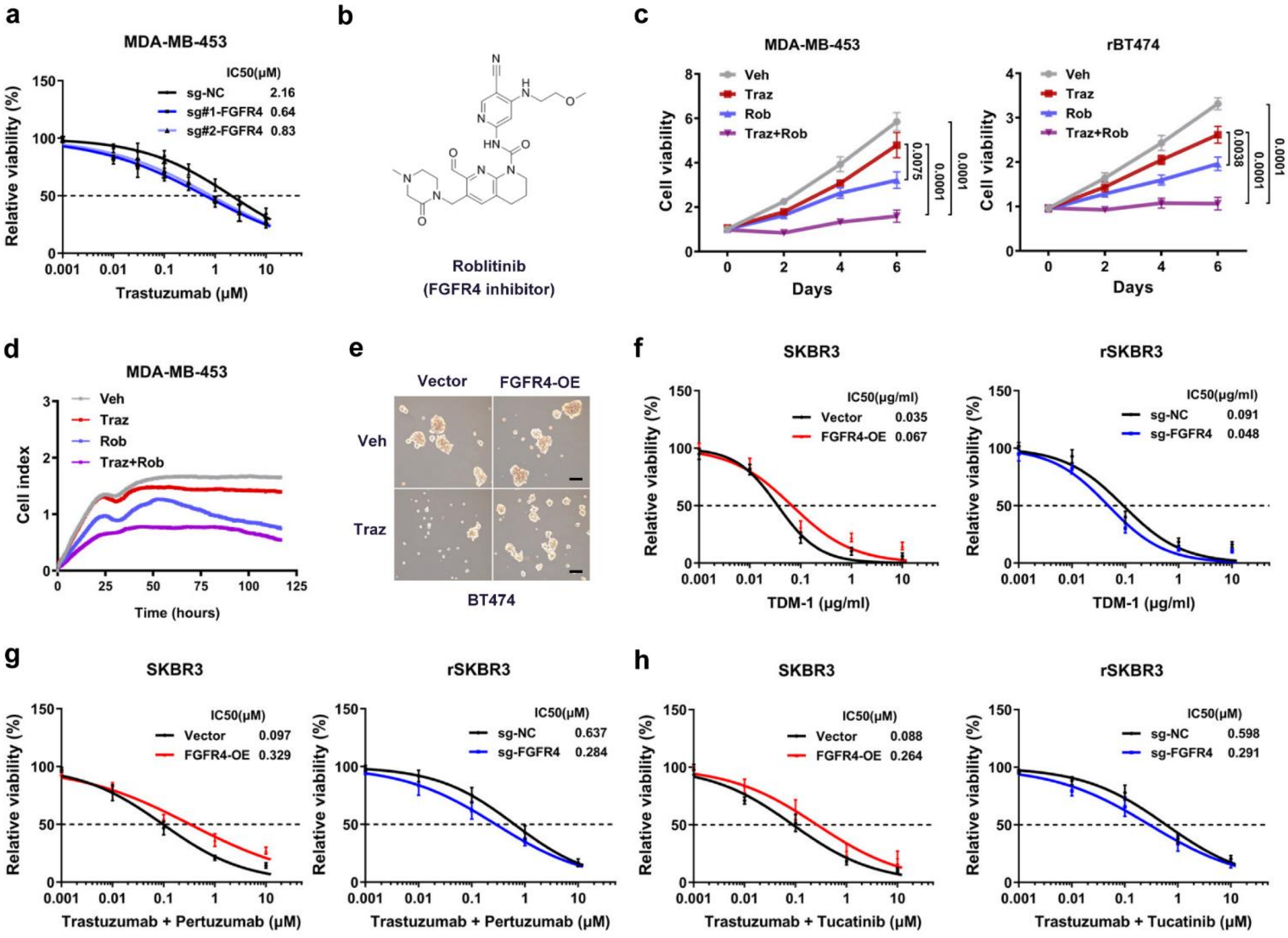

Supplementary Figure 3. FGFR4 inhibition confers sensitivity to anti-HER2 treatment in both intrinsic and acquired resistant cells.

(a) Dose-response curves of trastuzumab-resistant cells (MDA-MB-453) carrying sg-NC or sg-FGFR4 constructs after treated with trastuzumab. **(b)** Molecular structure of highly selective FGFR4 inhibitor roblitinib (also named as FGF-401). **(c)** Cell viability was measured in trastuzumab-sensitive cells treated with vehicle (Veh), 0.5 μM trastuzumab (Traz) and/or 0.5 μM roblitinib (Rob, FGFR4 inhibitor), respectively. Cell viability was measured using absorbance value at 450 nm, determined by CCK-8 assays. Group treated with vehicle (control) at day 0 was defined as 1 relative cell viability. Data were analyzed by one-way ANOVA adjusted for multiple comparisons. **(d)** Real-time monitoring of living cells was performed to evaluate the efficacy of trastuzumab and roblitinib in trastuzumab-resistant MDA-MB-453 cells. **(e)** Mammosphere formation assay revealed the effects of FGFR4 in protecting HER2-positive breast cancer stem cells from inhibiting by trastuzumab. Scale bar=100 μm. **(f)** Dose-response curves of sensitive cells (SKBR3) carrying vector or FGFR4-overexpression constructs after treated with TDM-1 (left). Dose-response curves of resistant cells (rSKBR3) carrying sg-NC or sg-FGFR4 constructs after treated with

TDM-1 (right). **(g)** Dose-response curves of sensitive cells (SKBR3) carrying vector or FGFR4-overexpression constructs after treated with combination of trastuzumab and pertuzumab at molar ratio of 1:1 (left). Dose-response curves of resistant cells (rSKBR3) carrying sg-NC or sg-FGFR4 constructs after treated with combination of trastuzumab and pertuzumab at molar ratio of 1:1 (right). **(h)** Dose-response curves of sensitive cells (SKBR3) carrying vector or FGFR4-overexpression constructs after treated with combination of trastuzumab and tucatinib at molar ratio of 10:1 (left). Dose-response curves of resistant cells (rSKBR3) carrying sg-NC or sg-FGFR4 constructs after treated with combination of trastuzumab and tucatinib at molar ratio of 10:1 (right). Cell viability was measured using absorbance value at 450 nm, determined by CCK-8 assays. Group treated with vehicle (control) was defined as 100% relative viability. Data in a, c, f, g, and h were presented as mean ± S.D., n = 3 biologically independent samples. Source data are provided as a Source Data file.

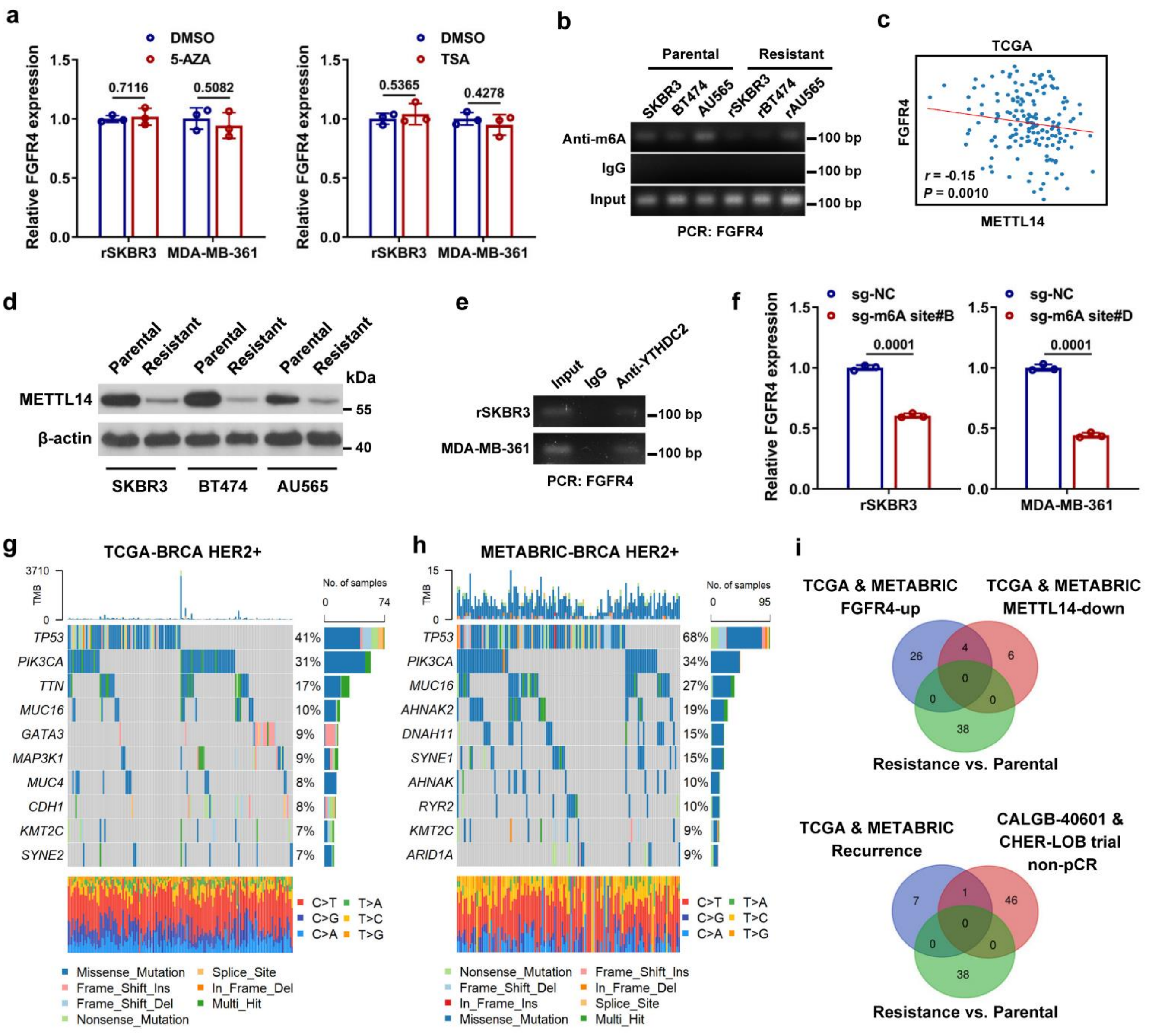

Supplementary Figure 4. m6A RNA hypomethylation mediates FGFR4 upregulation in trastuzumab-resistant HER2-positive breast cancer cells.

(a) *FGFR4* expression level was detected after treated with 5-Azacytidine (DNA methylation inhibitor) or Trichostatin A (HDAC inhibitor) in rSKBR3 and MDA-MB-361 cells. Data were analyzed by two-sided Student's t-test. **(b)** Agarose electrophoresis analysis of *FGFR4* mRNA PCR products after MeRIP assays in parental sensitive and resistant HER2-positive breast cancer cells. **(c)** Correlation between *FGFR4* and *METTL14* expression in TCGA database for HER2-positive breast cancer. Two-sided Spearman test was used. **(d)** Western blot assays showing that METTL14 expression were decreased in trastuzumab-resistant HER2-positive breast cancer cells. **(e)** Agarose electrophoresis analysis of FGFR4 mRNA PCR products after RIP assays by antibody against YTHDC2 in rSKBR3 and MDA-MB-361. **(f)** *FGFR4* expression level was detected after precise m6A modification in rSKBR3 and MDA-MB-361 cells. Data were analyzed by

two-sided Student's t-test. **(g-h)** Top ten frequently mutated genes of HER2-positive breast cancer in TCGA and METABRIC cohort. **(i)** Venn diagram presenting the number of mutated genes in resistant cell group as compared with other certain groups. Data in a and f were presented as mean ± S.D., n = 3 biologically independent samples. Source data are provided as a Source Data file.

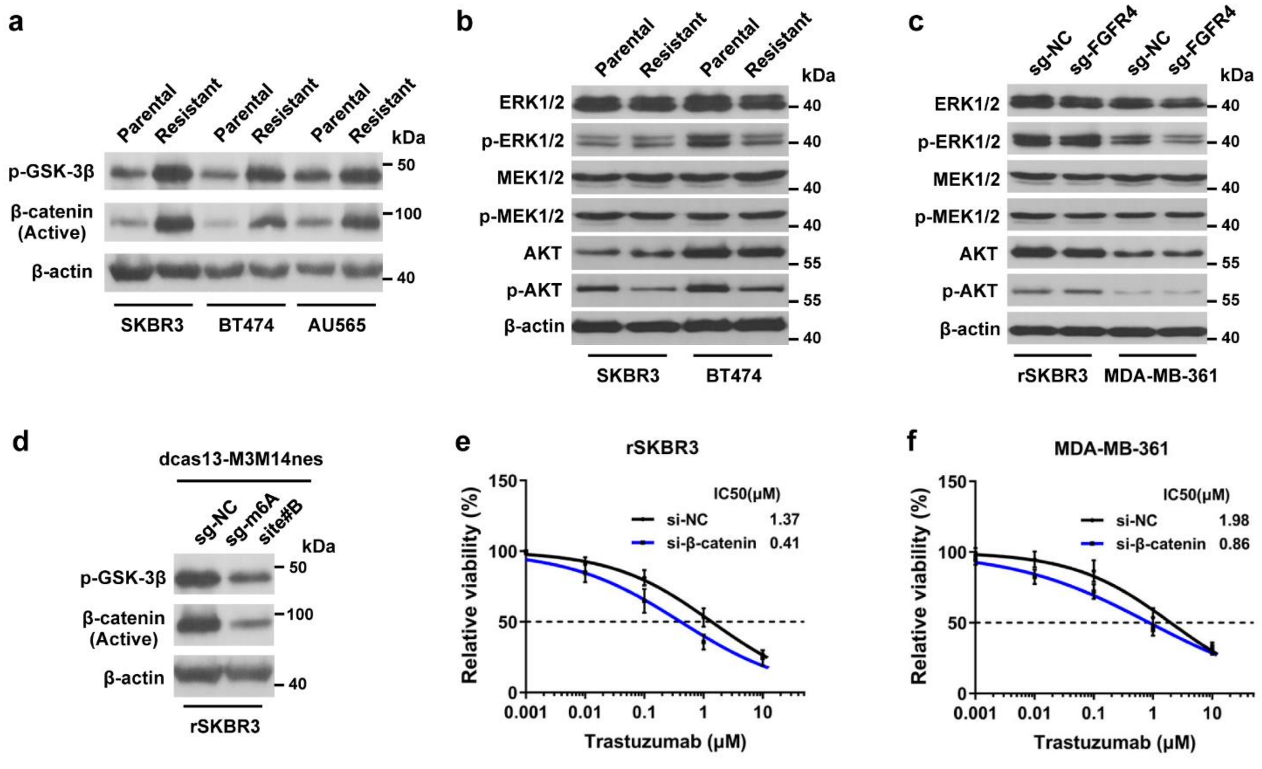

Supplementary Figure 5. FGFR4 activates β-catenin signaling to promote trastuzumab resistance in HER2-positive breast cancer.

(a) Western blot assays showing that β-catenin signaling were activated in trastuzumab-resistant cells compared to their parental trastuzumab-sensitive cells. **(b)** Western blot assays showing that MAPK and AKT signaling were attenuated in trastuzumab-resistant cells compared to their parental trastuzumab-sensitive cells. **(c)** Effect of the FGFR4 inhibition on the MAPK and AKT signaling pathway in rSKBR3 and MDA-MB-361 resistant breast cancer cells as assessed by western blot analysis. **(d)** dCas13b-METTL3/14 fusion system was used to precisely conduct m6A modification on *FGFR4* mRNA. Western blot assays revealed that β-catenin signaling were attenuated after increasing the m6A-modificated level of *FGFR4* mRNA in rSKBR3 breast cancer cells. **(e-f)** Dose-response curves of trastuzumab-resistant cells (rSKBR3 and MDA-MB-361) transfected with si-NC or si-β-catenin after treatment with trastuzumab. Cell viability was measured using absorbance value at 450 nm, determined by CCK-8 assays. Group treated with vehicle (control) was defined as 100% relative viability. Data in e and f were presented as mean ± S.D., n = 3 biologically independent samples. Source data are provided as a Source Data file.

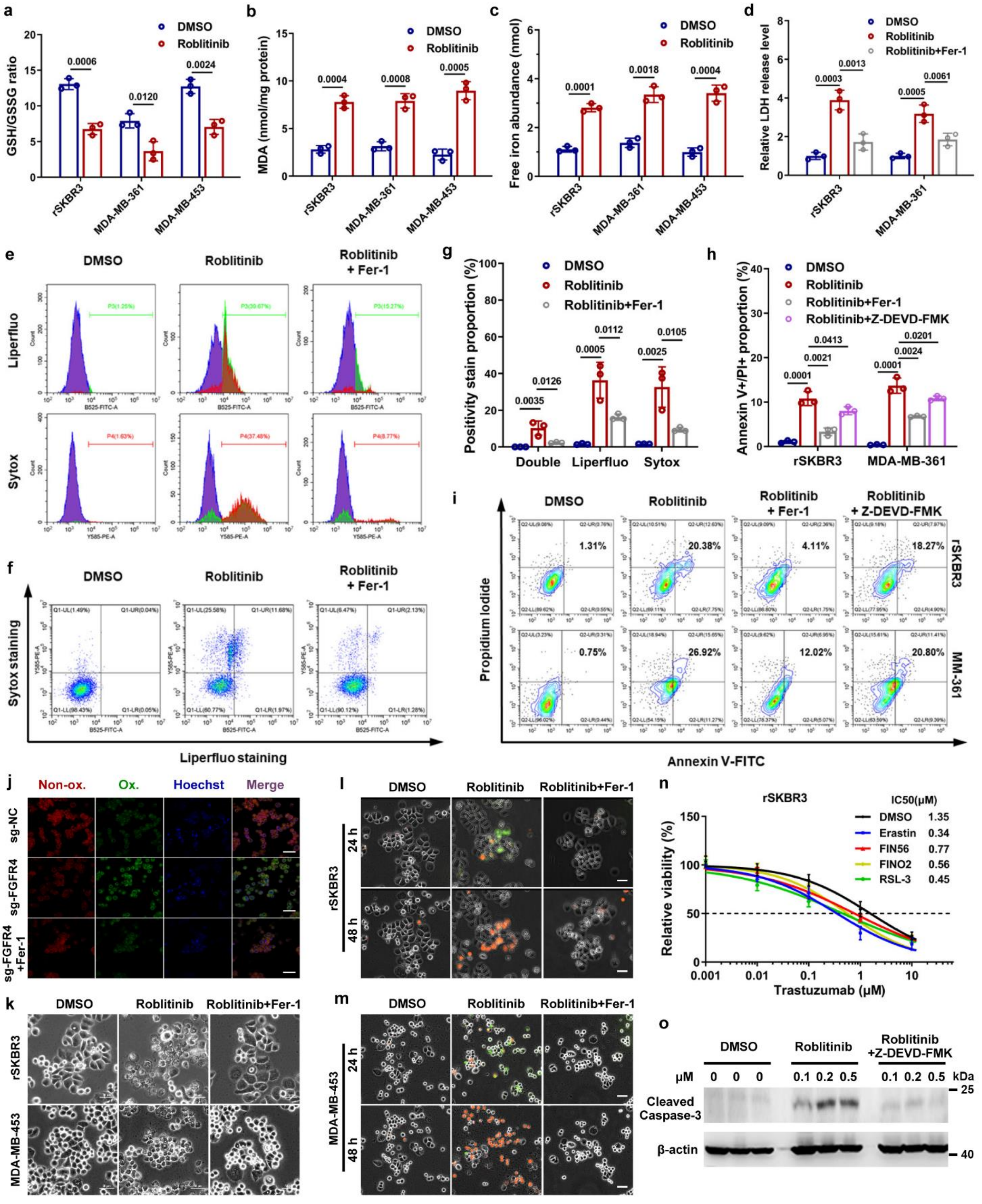

Supplementary Figure 6. Ferroptosis is triggered by FGFR4 inhibition in HER2-positive breast cancer.

(a) Glutathione (GSH) to oxidized glutathione (GSSG) ratio was evaluated in trastuzumab-resistant HER2-positive breast cancer cells. **(b)** The level of MDA (lipid peroxidation product) was significantly increased in roblitinib treated cells. **(c)** The level of intracellular ferrous ion (Fe^{2+}) was measured by colorimetric iron assays. **(d)** Cell death was determined by measuring LDH release in the culture supernatants. Presence of

Fer-1 (1 μM) rescued cells from death triggered by roblitinib (0.5 μM). **(e-g)** Flow cytometry assays were conducted after liperfluo and sytox staining to detect oxidized and dead cells, respectively. Liperfluo and sytox positivity rate was increased after roblitinib treatment (0.5 μM) and rescued by the presence of Fer-1 (1 μM). **(h-i)** Annexin V/PI staining showed that roblitinib induced cell death could be overwhelmingly reversed by Fer-1 (1 μM) and slightly reversed by Z-DEVD-FMK (a caspase-inhibitor, 10 μM). **(j)** C11-BODIPY probe staining showing that the ratio of oxidized to nonoxidized lipids was remarkably increased after FGFR4 inhibition and reversed by Fer-1 (1 μM), as assessed in MDA-MB-361 cells by confocal imaging. Scale bar=30 μm. **(k)** Phase-contrast photograph of rSKBR3 and MDA-MB-453 cells after roblitinib treatment to evaluate morphological changes before cell death. Nuclei and cytomembrane are intact before cell death which is the characteristics of ferroptosis. **(l-m)** Images show that lipid peroxide (marked by liperfluo, green) was accumulated before cell death (marked by sytox, red) in rSKBR3 and MDA-MB-453 cells treated with roblitinib. Scale bar=50 μm. **(n)** Four different classes of ferroptosis inducers were used to evaluate the impact of ferroptosis on trastuzumab resistance. Usage of Erastin (10 μM), FIN56 (1 μM), FINO2 (10 μM), and RSL-3 (1 μM) restored trastuzumab sensitivity in rSKBR3 breast cancer cells. Cell viability was measured using absorbance value at 450 nm, determined by CCK-8 assays. Group treated with vehicle (control) was defined as 100% relative viability. **(o)** Western blot assay showed cleaved caspase-3 was increased after roblitinib treatment and reduced by Z-DEVD-FMK (10 μM) in rSKBR3 breast cancer cells. Data in a-d, g, h, and n were presented as mean ± S.D., n = 3 biologically independent samples. Data were analyzed by two-sided Student's t-test in a-c, and one-way ANOVA adjusted for multiple comparisons in d, g, h. Source data are provided as a Source Data file.

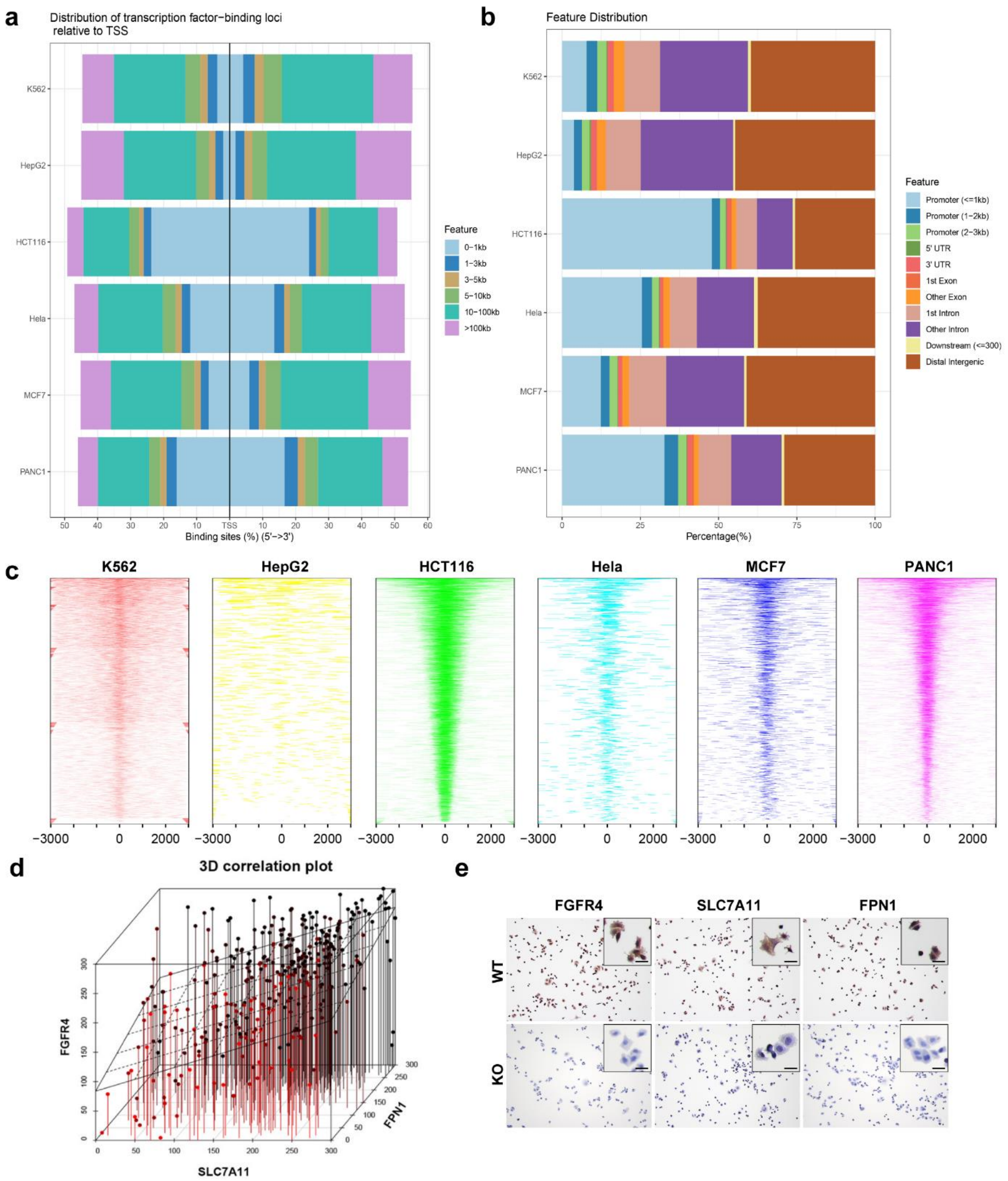

Supplementary Figure 7. Glutathione metabolism and Fe^{2+} transport is regulated by FGFR4 in HER2-positive breast cancer.

(a) Distribution of TCF-4 transcriptional factor-binding loci relative to TSS according to ChIP-seq data of ENCODE. **(b)** Distribution of TCF-4 transcriptional factor-binding loci of different elements according to ChIP-seq data of ENCODE. **(c)** Bioinformatic analysis of TCF-4 ChIP-seq of the ENCODE database indicates binding sites at promoter region. **(d)** Correlation between FGFR4, SLC7A11 and FPN1 protein expression in SYSUCC cohort showed by 3D plotter. **(e)** FGFR4, SLC7A11 and FPN1 IHC staining are confirmed to be specific by knockout negative controls in rSKBR3 breast cancer cells. Scale bar=20 μm.

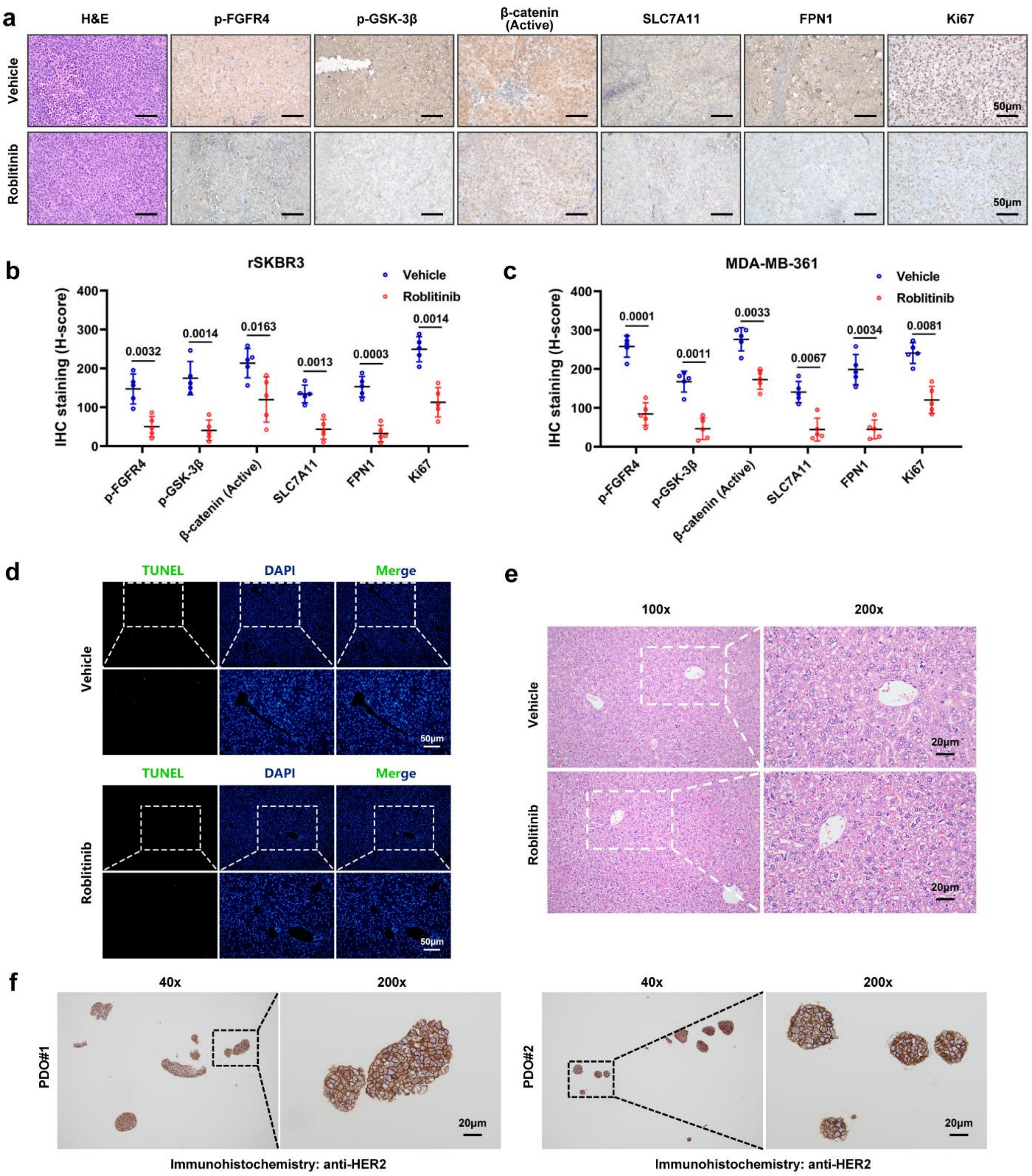

Supplementary Figure 8. Patient-derived models revealed the potent efficacy of FGFR4 inhibitor roblitinib with safety in trastuzumab-resistant HER2-positive breast cancer.

(a) Representative H&E and IHC staining images of rSKBR3 cell-based tumors from vehicle and roblitinib group. **(b-c)** Quantification of IHC staining by H-score method in vehicle and roblitinib group. Data were presented as mean ± S.D., n = 5 in each group. Data were analyzed by two-sided Student's t-test. **(d-e)** Detected by Tunel and H&E staining, roblitinib has no apparent toxicity to the liver at this dose which indicated the safety in clinical application. **(f)** HER2 expression was validated by IHC staining to confirm the origin of HER2-positive breast cancer organoids. Scale bar=20 μm.

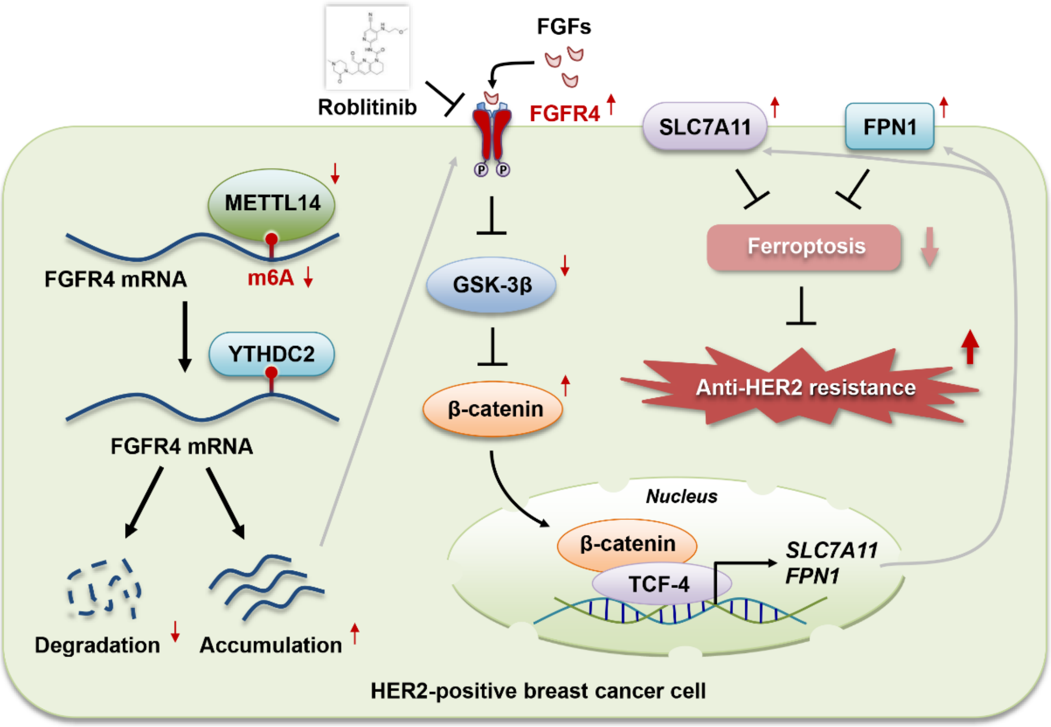

Supplementary Figure 9. Schematic diagram illustrates the mechanism of FGFR4 mediated anti-HER2 resistance in breast cancer.

N6-methyladenosine (m6A) modification level is reduced due to the downregulation of METTL14 in anti-HER2 resistant breast cancer. Decrease of m6A level prevents the YTHDC2 mediated *FGFR4* mRNA degradation, therefore, lead to the accumulation of FGFR4 in resistant breast cancer. FGFR4 phosphorylates GSK-3β and activates β-catenin/TCF4 signaling to increase the transcription of the *SLC7A11* and *FPN1* gene. Upregulated SLC7A11 and FPN1 accelerates glutathione synthesis and Fe^{2+} efflux, which confer anti-HER2 resistance by attenuating ferroptosis in breast cancer. Roblitinib, a highly selective inhibitor of FGFR4, overcomes anti-HER2 resistance by triggering ferroptosis in recalcitrant HER2-positive breast cancer.

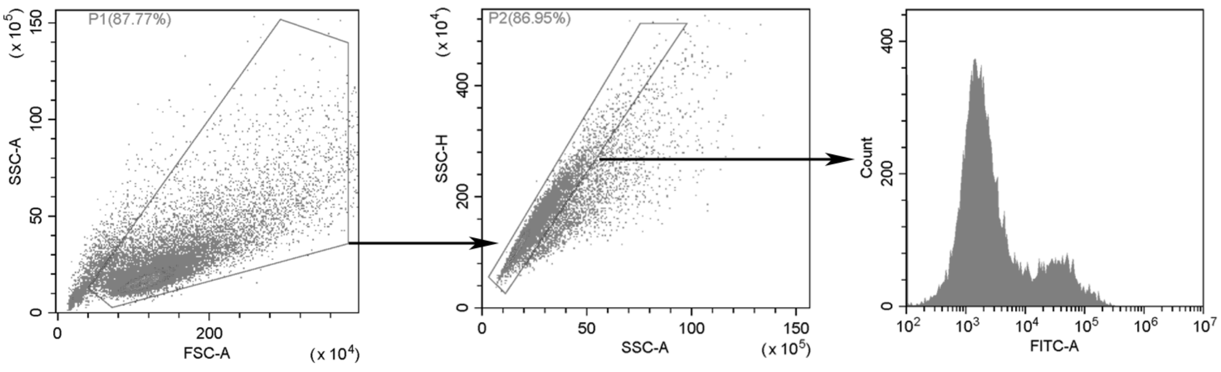

Supplementary Figure 10. Gating strategy of flow cytometric analysis.

Supplementary Table 1. Correlation of FGFR4 expression with clinicopathologic characteristics of HER2-positive breast cancer patients in SYSUCC.

Variables	Cases	FGFR4		*P* value
		Low	High	
Total	n=332	n=153	n=179	
**Age (y)**				
>50	157	65(41.4%)	29(58.6%)	
≤50	175	88(50.3%)	87(49.7%)	0.105
**Menopause**				
No	199	97(48.7%)	102(51.3%)	
Yes	133	56(42.1%)	77(57.9%)	0.234
**Family history**				
No	316	144(45.6%)	172(54.4%)	
Yes	16	9(56.2%)	7(43.8%)	0.403
**Histological grade**				
G1-2	194	98(50.5%)	96(49.5%)	
G3	138	55(39.9%)	83(60.1%)	0.055
**T status**				
T1-2	285	147(51.6%)	138(48.4%)	
T3-4	47	6(12.8%)	41(87.2%)	0.001*
**Lymph node status**				
Negative	163	93(57.1%)	70(42.9%)	
Positive	169	60(35.5%)	109(64.5%)	0.001*
**TNM stage**				
I-II	230	135(58.7%)	95(41.3%)	
III-IV	102	18(17.6%)	84(82.4%)	0.001*
**Recurrence**				
No	252	132(52.4%)	120(47.6%)	
Yes	80	21(26.2%)	59(73.8%)	0.001*

* $P < 0.05$, statistically significant. Data were analyzed by chi-squared test.

Supplementary Table 2. Univariate and multivariate Cox regression analysis of FGFR4 and relapse-free survival in patients with HER2-positive breast cancer patients in SYSUCC.

**Parameter**	**Univariate analysis**			**Multivariate analysis**		
	HR	**95% *CI***	***P***	***HR***	**95% *CI***	***P***
Age (≤50 vs. >50 years)	1.573	1.000-2.474	0.050	1.524	0.962-2.415	0.072
Menopause (Yes vs. No)	1.098	0.698-1.726	0.686	NA		
Histological grade (G3 vs. G1-2)	0.964	0.616-1.508	0.872	NA		
T status (T3-4 vs. T1-2)	2.880	1.761-4.710	0.001	1.359	0.764-2.418	0.297
Lymph node status (Positive vs. Negative)	3.016	1.846-4.929	0.001	1.680	0.946-2.983	0.077
TNM stage (III-IV vs. I-II)	4.761	3.024-7.496	0.001	2.635	1.432-4.850	0.002*
FGFR4 expression (High vs. Low)	2.836	1.723-4.670	0.001	1.772	1.028-3.054	0.039*

NA: not analyze; * P <0.05, statistically significant. Data were analyzed by two-sided log-rank test.

Supplementary Table 3. Univariate and multivariate Cox regression analysis of FGFR4 and overall survival in patients with HER2-positive breast cancer patients in SYSUCC.

Parameter	Univariate analysis			Multivariate analysis		
	HR	**95% *CI***	***P***	***HR***	**95% *CI***	***P***
Age (≤50 vs. >50 years)	1.657	0.985-2.787	0.057	NA		
Menopause (Yes vs. No)	1.283	0.758-2.172	0.353	NA		
Histological grade (G3 vs. G1-2)	0.739	0.433-1.260	0.267	NA		
T status (T3-4 vs. T1-2)	2.917	1.684-5.051	0.001	1.441	0.760-2.730	0.263
Lymph node status (Positive vs. Negative)	3.756	2.071-6.815	0.001	1.672	0.906-3.088	0.100
TNM stage (III-IV vs. I-II)	4.458	2.662-7.467	0.001	2.196	1.117-4.317	0.023*
FGFR4 expression (High vs. Low)	2.794	1.582-4.937	0.001	2.331	1.185-4.586	0.014*

NA: not analyze; * P <0.05, statistically significant. Data were analyzed by two-sided log-rank test.

Supplementary Table 4. High impact mutation genes between resistant and parental HER2-positive SKBR3 breast cancer cell.

Chr	Pos	Ref	Alt	Gene_Name	Annotation	Gene_ID	trans_ID
1	16620040	T	C	CROCCP2	splice_acceptor_variant&intron_variant	ENSG00000215908	ENST00000640476.1
1	22027781	G	C	LINC00339	splice_donor_variant&intron_variant	ENSG00000218510	ENST00000634451.2
1	26553429	T	C	RPS6KA1	stop_lost	ENSG00000117676	ENST00000374163.5
1	37864334	A	AT	INPP5B	frameshift_variant	ENSG00000204084	ENST00000373024.7
1	148105470	C	CATCT	NBPF11	frameshift_variant	ENSG00000263956	ENST00000615281.4
1	156727416	C	CT	ISG20L2	frameshift_variant	ENSG00000143319	ENST00000313146.10
2	130155147	G	A	SMPD4	stop_gained	ENSG00000136699	ENST00000409031.5
2	36578663	C	CT	FEZ2	frameshift_variant	ENSG00000171055	ENST00000379245.8
2	118108329	C	CA	INSIG2	frameshift_variant	ENSG00000125629	ENST00000614681.1
2	130931184	GAA	G	ARHGEF4	frameshift_variant	ENSG00000136002	ENST00000525839.6
3	142163824	T	C	GK5	splice_acceptor_variant&intron_variant	ENSG00000175066	ENST00000492097.5
3	198186516	T	C	FAM157A	splice_donor_variant&intron_variant	ENSG00000236438	ENST00000634862.1
3	129436658	G	GT	MBD4	frameshift_variant	ENSG00000129071	ENST00000249910.5
5	140524127	G	GA	ANKHD1	frameshift_variant	ENSG00000131503	ENST00000360839.6
6	31271816	T	C	HLA-C	splice_donor_variant&intron_variant	ENSG00000204525	ENST00000640219.1
6	108051166	A	AT	OSTM1	frameshift_variant	ENSG00000081087	ENST00000193322.7
7	30572998	A	G	AC005154.1	splice_donor_variant&intron_variant	ENSG00000196295	ENST00000584621.5
7	32755409	C	CT	DPY19L1P1	splice_acceptor_variant&intron_variant	ENSG00000229358	ENST00000417811.2
8	85219234	A	T	C8orf59	splice_donor_variant&intron_variant	ENSG00000176731	ENST00000612977.4
8	123254534	C	CT	ZHX1	frameshift_variant	ENSG00000165156	ENST00000395571.7
9	35757196	T	C	AL133410.2	splice_acceptor_variant&intron_variant	ENSG00000228843	ENST00000425499.2
10	50745132	T	C	ASAH2B	start_lost	ENSG00000204147	ENST00000374007.5
12	123584638	T	A	TMED2	start_lost	ENSG00000086598	ENST00000262225.7
14	64093374	G	A	SYNE2	stop_gained	ENSG00000054654	ENST00000344113.8
15	101972594	A	G	WASH3P	splice_acceptor_variant&intron_variant	ENSG00000185596	ENST00000354296.9
16	30010902	C	G	DOC2A	splice_donor_variant&intron_variant	ENSG00000149927	ENST00000350119.8
16	79211976	G	GGTAAA	WWOX	frameshift_variant	ENSG00000186153	ENST00000402655.6
17	46148241	C	T	KANSL1	splice_acceptor_variant&intron_variant	ENSG00000120071	ENST00000639356.1
17	44215911	G	GT	UBTF	frameshift_variant	ENSG00000108312	ENST00000526094.5
18	5570219	T	C	AP005059.1	splice_donor_variant&intron_variant	ENSG00000264000	ENST00000578391.1
19	38733773	A	G	CAPN12	stop_lost	ENSG00000182472	ENST00000594552.5
19	48481921	TA	T	CYTH2	frameshift_variant&splice_region_variant	ENSG00000105443	ENST00000620214.1
20	62387999	T	C	RPS21	stop_lost	ENSG00000171858	ENST00000370592.2
21	36137393	T	C	CBR3-AS1	splice_acceptor_variant&intron_variant	ENSG00000236830	ENST00000625079.3
21	34385627	G	GA	SMIM11A	frameshift_variant	ENSG00000205670	ENST00000399295.2
22	42140302	G	A	CYP2D7	stop_gained	ENSG00000205702	ENST00000433992.2
X	156021692	A	G	WASH6P	splice_acceptor_variant&intron_variant	ENSG00000182484	ENST00000476066.6
X	118680640	C	CA	DOCK11	frameshift_variant	ENSG00000147251	ENST00000633080.1

Supplementary Table 5. Sequence of primers used in this study.

Primers for qRT-PCR assay.

FGFR4	Forward	GAGGGGCCGCCTAGAGATT
	Reverse	CAGGACGATCATGGAGCCT
SLC7A11	Forward	TCTCCAAAGGAGGTTACCTGC
	Reverse	AGACTCCCCTCAGTAAAGTGAC
FPN1	Forward	TGGATGGGTTCTCACTTCCTG
	Reverse	GTCAATCCTTCGTATTGTGGCAT
GPX4	Forward	GAGGCAAGACCGAAGTAAACTAC
	Reverse	CCGAACTGGTTACACGGGAA
METTL3	Forward	TTGTCTCCAACCTTCCGTAGT
	Reverse	CCAGATCAGAGAGGTGGTGTAG
METTL14	Forward	AGTGCCGACAGCATTGGTG
	Reverse	GGAGCAGAGGTATCATAGGAAGC
METTL16	Forward	TTCTGTCAAGGTCGGACAATG
	Reverse	CAGCACCACGAATGTTATGGG
RBM15	Forward	GTGAGGACTCGACTTCCCG
	Reverse	GCCGCTATCGGTCTTTCCG
VIRMA	Forward	TACTTTGAGCCCATTTCTCCTGA
	Reverse	GGAATACTGTCTACTGTTCGTCG
WTAP	Forward	CTTCCCAAGAAGGTTCGATTGA
	Reverse	TCAGACTCTCTTAGGCCAGTTAC
ALKBH5	Forward	CGGCGAAGGCTACACTTACG
	Reverse	CCACCAGCTTTTGGATCACCA
FTO	Forward	ACTTGGCTCCCTTATCTGACC
	Reverse	TGTGCAGTGTGAGAAAGGCTT
YTHDC2	Forward	CAAAACATGCTGTTAGGAGCCT
	Reverse	CCACTTGTCTTGCTCATTTCCC
β-actin	Forward	GAAATCGTGCGTGACATTAA
	Reverse	AAGGAAGGCTGGAAGAGTG

Primers for PCR after ChIP assay.

**_SLC7A11_ promotor**		
-2000~-1750bp	Forward	ACATGAAAGTACAATCCTTTTGGT
	Reverse	GCCAAAGACCCCACTCAGATA
-1750~-1500bp	Forward	TGTTCCCTGAGATTCCTAGTGTC
	Reverse	GATTAATGGACCTAAGGAGCAGC
-1500~-1250bp	Forward	AGGTGGTGTCATTTTCTCAGGT

	Reverse	ATGGTGAGGATGATCAAAGAAAGAA
-1250~-1000bp	Forward	GCGTATAAAGGTAGCTTCAGGGT
	Reverse	CCCTTCTAACTTTCTCAACCAAGTG
-1000~-750bp	Forward	TGTTTTCCTTTACAGGCTTTTGCT
	Reverse	TGTCATGTTGTTCCTTTGAATTACC
-750~-500bp	Forward	TGACTATTTTCTGGAGTCATGGTGA
	Reverse	CACACAACTATAAGCCTTCCTCAA
-500~-250bp	Forward	TCTACTCACAAAACAGTCGCA
	Reverse	AGCAACTCGTAGTGAGCAACA
-250~0bp	Forward	AGCTGAGTAATGCTGGAGGC
	Reverse	CTCAGCTTCCTCATGGGCTT
***FPN1* promotor**		
-2000~-1750bp	Forward	CCACAGCTCACTCACTGGTT
	Reverse	TCACCAAGGAGTCCCCAGAA
-1750~-1500bp	Forward	GCTGACTTAGCCACTCTTTCCA
	Reverse	TCATGCTGTCCCCAACTTCC
-1500~-1250bp	Forward	GATCGCCATAAAGTGGAGGG
	Reverse	AGGTTTTGGTTTGCTAACCGTG
-1250~-1000bp	Forward	CACATACTCATGCGTGGCTC
	Reverse	CACGAAAACCCTGGTAGTGG
-1000~-750bp	Forward	TGGTTTGGCACAGCAGGATT
	Reverse	TCCCTGGATCTGTCCCTGTC
-750~-500bp	Forward	GGGGATACGGGTAGGTCTGT
	Reverse	GGGCTTCCCTCAAAGAACCA
-500~-250bp	Forward	CTGGAGCTTTGCACTGCGAC
	Reverse	CTGCGCGAAACTAAGCGTC
-250~0bp	Forward	GGAGTGGAACGCGTCGAG
	Reverse	ACGAGCTCCCGTCAACCT

Supplementary Table 6. Antibodies used in this study.

Antibody	Source	Application
FGFR4	SANTA CRUZ	1:1000 for WB; 1:200 for IHC; 1:400 for IF
GSK-3β	Cell Signaling Technology	1:1000 for WB
p-GSK-3β	Cell Signaling Technology	1:1000 for WB; 1:50 for IHC; 1:100 for IF
β-catenin	Cell Signaling Technology	1:1000 for WB; 1:800 for IHC; 1:1600 for IF; 1:100 for ChIP
ERK1/2	Cell Signaling Technology	1:1000 for WB
p-ERK1/2	Cell Signaling Technology	1:2000 for WB
MEK1/2	Cell Signaling Technology	1:1000 for WB
p-MEK1/2	Cell Signaling Technology	1:1000 for WB
AKT	Cell Signaling Technology	1:1000 for WB
p-AKT	Cell Signaling Technology	1:2000 for WB
HER2	Cell Signaling Technology	1:200 for IHC
TCF-4	Cell Signaling Technology	1:50 for ChIP
SLC7A11	Cell Signaling Technology	1:1000 for WB; 1:200 for IHC; 1:400 for IF
FPN1	Novus Biologicals	1:1000 for WB; 1:200 for IHC; 1:400 for IF
GPX4	Abcam	1:1000 for WB
TFRC	Thermofisher	1:1000 for WB
ACSL4	SANTA CRUZ	1:500 for WB
Ki67	Abcam	1:400 for IHC
β-tubulin	Affinity	1:3000 for WB
Histone H3	Affinity	1:1000 for WB
Cleaved Caspase-3	Cell Signaling Technology	1:1000 for WB
N6-methyladenosine	Synaptic Systems	1:1000 for Dotblot; 1:200 for IF; 5ug for IP
YTHDC2	Abcam	5ug for IP
METTL14	Abcam	1:500 for WB
β-actin	Cell Signaling Technology	1:2000 for WB

Supplementary Table 7. Sequence of sgRNAs used in this study.

FGFR4 (dCas9-KRAB)	sgRNA#1	GAGGAATGTACCCGCGACGG
	sgRNA#2	GCGGGTACATTCCTCGCTCC
	sgRNA#3	GACAGGAGGTGGGCCGCTCG
	sgRNA#4	AGCGAGGAATGTACCCGCGA
	sgRNA#5	GCGGGTACATTCCTCGCTCC
	sgRNA-NC	GGGCGAGGAGCTGTTCACCG
FGFR4 (dCas13b-M3M14nes)	sgRNA#siteB	TTAGGACTTGCACATAGGGGAAACCGTCGG
	sgRNA#siteD	CCAGCACATTGCGGGCAGCCAGGTCCCGGT
	sgRNA-NC	GTAATGCCTGGCTTGTCGACGCATAGTCTG

Supplementary Table 8. Sequence of mutated m6A site in this study.

Mutation site	Wild type	Mutant
Site #A	5’-CGCTATAACTACCTG-3’	5’-CGCTATATCTACCTG-3’
Site #B	5’-ACTGCAGACATCAAT-3’	5’-ACTGCAGTCATCAAT-3’
Site #C	5’-CCCCGGGACAGGCTG-3’	5’-CCCCGGGTCAGGCTG-3’
Site #D	5’-GGCATGGACCCTGCC-3’	5’-GGCATGGTCCCTGCC-3’

Supplementary Table 9. Databases analyzed in this study.

Dataset	Source	Identifier
The Cancer Genome Atlas	https://portal.gdc.cancer.gov/	BRCA
METABRIC	https://www.cbioportal.org/	BRCA
Cancer Cell Line Encyclopedia	https://portals.broadinstitute.org/ccle/	CCLE
Cancer Therapeutics Response Portal	https://portals.broadinstitute.org/ctrp/	CTRP
Metascape	https://metascape.org/	None
KM Plotter	https://kmplot.com/analysis/	None
CTGS	https://ctgs.biohackers.net/	None
m6A-Atlas	https://180.208.58.66/m6A-Atlas/index.html	None
JASPAR	https://jaspar.genereg.net/	TCF7L2
ENCODE	https://www.encodeproject.org/	TCF7L2
UCSC	https://genome.ucsc.edu/	None
DAVID	https://david.ncifcrf.gov/home.jsp/	None
