# Supplementary material for: N6-methyladenosine regulated FGFR4 attenuates ferroptotic cell death in recalcitrant HER2-positive breast cancer
Source: Nat Commun. 2022 May 13;13:2672. doi: 10.1038/s41467-022-30217-7 (PMC9106694; doi:10.1038/s41467-022-30217-7)
Supplement: Supplementary file 3 — Description of Additional Supplementary Files [file 41467_2022_30217_MOESM3_ESM.pdf]

Description of Additional Supplementary Files

Supplementary Movie 1. Time lapse video recorded the morphological changes before cell death after FGFR4 inhibition in rSKBR3 breast cancer cells.

Supplementary Movie 2. Time lapse video recorded the morphological changes before cell death after FGFR4 inhibition in MDA-MB-453 breast cancer cells.

Supplementary Movie 3. Time lapse video recorded the lipid peroxide accumulation (marked by liperfluo, green) and cell death (marked by sytox, red) after FGFR4 inhibition in rSKBR3 breast cancer cells.

Supplementary Movie 4. Time lapse video recorded the lipid peroxide accumulation (marked by liperfluo, green) and cell death (marked by sytox, red) after FGFR4 inhibition in MDA-MB-453 breast cancer cells.
